# Examining smoking-induced differential gene expression changes in buccal mucosa

**DOI:** 10.1186/1755-8794-3-24

**Published:** 2010-06-24

**Authors:** Doris M Kupfer, Vicky L White, Marita C Jenkins, Dennis Burian

**Affiliations:** 1 Civil Aerospace Medical Institute, Federal Aviation Administration, Oklahoma City, Oklahoma, USA; 2 Advancia, Inc., Oklahoma City, OK, USA

## Abstract

**Background:**

Gene expression changes resulting from conditions such as disease, environmental stimuli, and drug use, can be monitored in the blood. However, a less invasive method of sample collection is of interest because of the discomfort and specialized personnel necessary for blood sampling especially if multiple samples are being collected. Buccal mucosa cells are easily collected and may be an alternative sample material for biomarker testing. A limited number of studies, primarily in the smoker/oral cancer literature, address this tissue's efficacy as an RNA source for expression analysis. The current study was undertaken to determine if total RNA isolated from buccal mucosa could be used as an alternative tissue source to assay relative gene expression.

**Methods:**

Total RNA was isolated from swabs, reverse transcribed and amplified. The amplified cDNA was used in RT-qPCR and microarray analyses to evaluate gene expression in buccal cells. Initially, RT-qPCR was used to assess relative transcript levels of four genes from whole blood and buccal cells collected from the same seven individuals, concurrently. Second, buccal cell RNA was used for microarray-based differential gene expression studies by comparing gene expression between a group of female smokers and nonsmokers.

**Results:**

An amplification protocol allowed use of less buccal cell total RNA (50 ng) than had been reported previously with human microarrays. Total RNA isolated from buccal cells was degraded but was of sufficient quality to be used with RT-qPCR to detect expression of specific genes. We report here the finding of a small number of statistically significant differentially expressed genes between smokers and nonsmokers, using buccal cells as starting material. Gene Set Enrichment Analysis confirmed that these genes had a similar expression pattern to results from another study.

**Conclusions:**

Our results suggest that despite a high degree of degradation, RNA from buccal cells from cheek mucosa could be used to detect differential gene expression between smokers and nonsmokers. However the RNA degradation, increase in sample variability and microarray failure rate show that buccal samples should be used with caution as source material in expression studies.

## Background

Blood has been shown to be a responsive tissue that is useful for monitoring gene expression changes due to disease, environmental, biological or drug effects. However, for studies performed in human subjects, a less invasive tissue source for biomarker monitoring is of interest due to the discomfort, required skill level, and cost of blood collection, especially for repeated-measures studies. Buccal mucosa (from cheek swabs) is an easily accessed tissue and has been used successfully to obtain DNA for genotyping studies [1]. However, the literature is limited as to the usefulness of RNA from buccal cells as a substrate for gene expression testing, presumably due to concern regarding a high concentration of RNases in saliva which are known to rapidly degrade RNA in these cells [2]. qPCR has been used to detect expression changes in genes from the P450 family using snap frozen surgical buccal plug samples [3] and from brushed exfoliated buccal cells [4,5]. These studies suggested that buccal cells might serve as an alternative to blood in qPCR assays examining gene expression profiles after exposure to environmental toxins, tobacco smoke, drugs, nutrients, or the presence of certain cancers. With RNA purified from brushed exfoliated buccal cells, Sridhar et al. [6] used microarrays from smoker samples they collected and nonsmoker arrays from the Gene Expression Omnibus (GEO) collection to compare expression levels between smokers and nonsmokers, and to compare expression patterns between buccal cells and bronchial epithelium in smokers and nonsmokers from an earlier microarray-based study [7] by Gene Set Enrichment Analysis (GSEA) [8]. To our knowledge, buccal cells have not been used in a whole transcriptome approach to investigate differential gene expression between smokers and nonsmokers using concurrently harvested samples in a manner which directly compares expression differences. A successful study of this type would more clearly suggest that buccal cells have efficacy as source material for biomarker discovery or in a gene expression monitoring system than earlier studies.

We describe here, both qPCR and microarray approaches. The RT-qPCR study used matched blood and brushed buccal samples from the same subjects. Relative expression levels of four genes allowed comparison of tissue sources and subject differences. RNA from buccal cells was highly degraded; nonetheless, expression could be detected by qPCR for all four transcripts tested. This was sufficient evidence of the potential of buccal cells to follow up on the work of Sridhar et al. [6] and use microarrays for differential gene expression analysis on the transcriptome level in smokers and nonsmokers. An important consideration was the availability of the Smoking Induced Epithelial Gene Expression Database, (SEIGE) [7] and smoker buccal mucosa-specific gene lists [6] against which results from this study could be compared for confirmation of our method.

Our data was first analyzed for differences between smokers and nonsmokers using Significance Analysis of Microarray (SAM) [9] and Rank Product (RP) [10] for detection of significant gene expression differences between smokers and nonsmokers in our study. These analyses resulted in a list of candidate marker genes from each method. Ingenuity Pathway Analysis [11] was used to find functional networks containing the differentially expressed genes. The gene lists were also examined for transcriptional coregulation by searching the promoters of differentially expressed genes for transcription factor binding sites (TFBS) using PAINT [12] to access the TRANSFAC [13] database of known TFBS. Specifically, we identified 103 genes with RP analysis that had increased expression in smokers. Pathway analysis showed five function networks involving 91 of the 103 target genes. Network functions included cell cycle; cell growth, proliferation and movement; gene expression; and immunological disease. Upstream sequence analysis showed 38 target genes containing binding sites for at least one of three widely expressed transcription factors. Twenty-five genes were identified using SAM analysis. Similar to the RP results, 13 of these genes fell into one of two functional networks which had in common roles in tumor morphology, metabolic disease, lipid and carbohydrate metabolism and which contained binding sites for at least one of two widely expressed transcription factors. These results suggest that many of these genes are co-regulated and that the transcriptional response affects numerous cellular functions. Both gene lists were further analyzed using GSEA, to compare the buccal dataset against the Sridhar gene sets. The comparisons showed that the genes in our buccal array data changed expression in the same direction as in the published sets.

The results of the study suggest that buccal mucosa may indeed be useful for factors selected carefully for optimum expression change in buccal tissue. However, the random degradation which may vary between subjects that we encountered suggests a loss of sensitivity, and possibly the need for multiple sampling which is costly. It also suggests that due to the extensive degradation found it seems unlikely to be a reliable source for biomarker discovery.

## Methods

### Sample Collection

All sample collection was performed with the informed consent of the study participants under the auspices of the Federal Aviation Administration Internal Review Board for approved protocol 08011. Seven subjects provided matched blood and buccal samples for the qPCR portion of the study and eight additional female subjects provided samples for the smoker vs. nonsmoker microarray study. Blood samples were collected in PAXgene™; Blood RNA tubes (PreAnalytix/Qiagen, Valencia, CA) according to the manufacturer's published protocol. Urine samples for nicotine and cotinine testing were collected in urine cups without preservative and refrigerated until shipping to a clinical lab (Diagnostic Laboratory of Oklahoma, Oklahoma City, OK). All nonsmokers were below the level of detection for both nicotine (10 ng/ml) and cotinine (40 ng/ml). All smokers showed levels above those expected for smokers which are concentrations greater than 100 ng/ml for nicotine and 200 ng/ml for cotinine. See Table 1 for this and demographic data.

**Table 1:** Smoker vs. nonsmoker demographics and nicotine/cotinine levels

Subjects	Group	Age	Ethnicity	Nicotine ng/ml	Cotinine ng/ml
NS21	nonmoker	41	cau	ND	ND
NS22	nonsmoker	44	cau	ND	ND
NS23	nonsmoker	27	cau	ND	ND
NS24	nonsmoker	53	cau	ND	ND
SM25	smoker	53	cau	990	900
SM26	smoker	47	cau	> 2500	980
SM27	smoker	39	cau	510	2100
SM28	smoker	50	his	> 2500	1000
11Sm*	smoker	50	his	> 2500	1000
12NS*	nonsmoker	44	cau	ND	ND

Shown is the demography of the female study population used in the microarray study. No statistical significance was found when ages of smokers and non-smokers were compared (see results). Also shown are the nicotine and cotinine levels for each subject (see methods).

* 11Sm and 12NS represent repeat sampling of subjects Sm28 and NS22, respectively. (cau, Caucasian; his, Hispanic; ND, not detected)

Buccal samples were collected using sterile Cytobrush Plus^® ^(Medscand Medical; Guttenberg, NJ). Subjects were asked not to eat for the 30 minutes prior to sampling and rinsed their mouths with a minimum of 20 ml of water before sample collection. Two buccal samples were collected from each subject and processed separately as either "a" or "b" samples. Cheeks were brushed for 30 seconds, and the brushes were immediately plunged into 2 ml tubes containing 1.0 ml of room temperature RNAlater (Invitrogen, Carlsbad, CA) to prevent post-sampling degradation of the RNA. The brush ends were cut off with sterile surgical scissors such that the 2 ml tubes could be capped. RNA was purified from buccal cell swabs immediately after collection.

### RNA Purification

RNA isolation from blood samples was performed according to the protocol in the PAXgene™; Blood RNA Purification Kit [14] with the optional on-column DNase treatment. A blood total RNA control sample was created by pooling purified RNA samples from three individuals not participating in either study.

Buccal-cell RNA was purified using the RNeasy Micro Kit (Qiagen, Valencia, CA) with the modifications found in Spivack et al. [5] and here. Cells were pelleted by centrifugation at 4,000 × g. The brush was removed from the tube by scraping the bristles against the lip of the tube to remove any adhered cells and the pellet reformed by centrifugation as above. RNAlater was pipetted off the pellet and the pellet washed with ice-cold PBS and the PBS removed after centrifugation, as above. Two microliters of polyC (Sigma Chemical; St. Louis MO) and 350 μl Buffer RLT (RNeasy Micro Kit) containing 10 μl/ml beta-mercaptoethanol was added and the pellet passed through a 25 ga needle to lyse the cells. The lysate was centrifuged at 20,000 × g for 3 minutes and the supernatant transferred to a fresh microfuge tube. Then 350 μl 70% ethanol was added, mixed well by pipetting and the sample applied to a MinElute column (RNeasy Micro Kit), and centrifuged at 8000 × g for 30 seconds. The column was washed twice with 350 μl of RW1 buffer (RNeasy Kit) followed by centrifugation at 8000 × g for 15 seconds. The column was placed in a fresh 2 ml collection tube and 500 ul RPE buffer (RNeasy Micro Kit) was added. The column was centrifuged at 8000 × g for 30 seconds. 500 μl of freshly prepared 80% ethanol was added the column and centrifuged for 2 minutes at 8000 × g. The column was transferred to a fresh 2 ml collection tube, with the cap open and centrifuged at 16,000 × g for 5 minutes. The RNA was eluted by adding 30 μl pre-warmed (50-55 degC) RNase-free water to the membrane. After 2 minutes incubation the column was centrifuged at 16,000 × g for 2 minutes. Spectrophotometric analysis showed a large 230 nm component, potentially salt carryover. To reduce this, the Qiagen RNeasy Micro Handbook RNA Cleanup and Concentration protocol (December 2007) was used as written by the manufacturer for sample volumes less than 100 μl.

RNA quality was assessed from Agilent Bioanalyzer 2100 (Agilent, Santa Clara, CA) traces using the Agilent RNA 6000 Nano Series II kit following manufacturer's directions with 1 μl of sample to generate a RNA Integrity Number (RIN). Yield was determined on a Nanodrop 1000 spectrophotometer (Thermo Scientific, Waltham, MA) (Additional file 1). RNA was aliquoted and stored at -80 degC.

### qPCR

Primers for qPCR in the matched blood and buccal portion of the study were designed using Beacon Designer 7.0 (PREMIER Biosoft International, Palo Alto, CA). Primers were synthesized and HPLC purified (Integrated DNA Technologies, Coralville IA). For three genes, integrin alpha-5/beta-1 (ITGA5), ankyrin repeat domain 28 (ANKRD28), and transmembrane protein 8 (TMEM8), multiple sets of primers were designed to span the mRNA. For ribosomal protein S3A, (RPS3A) only a single primer set was designed due to the small size of the transcript. See Additional file 2 for the primer sequences, positions of the primer sets on the respective transcript, concentrations and annealing temperatures. Template material for qPCR was prepared from 50 ng aliquots of total RNA that were reverse transcribed and amplified using either the WT-Ovation™ Pico System or the Ovation™ RNA Amplification System V2, #3300, 3100, respectively (Nugen Technologies, Inc., San Carlos, CA). All qPCR reactions were 25 μl and performed in triplicate with a SYBR^® ^green based based assay, PerfeCta SYBR Green FastMix, Low ROX, #95074-05k (Quanta Biosciences, Gaithersburg, MD) with no additional magnesium using 1 ng of amplified template material per reaction except in the amplification comparison series where 5 ng/reaction was used. Cycling was performed on a Stratagene MX3005p (Agilent Technologies, La Jolla, CA) in a 96-well polypropylene plate using optical strip caps (#410098 and 401425 Agilent Technologies). Cycling parameters were one cycle of 2 minutes 95 degC, 40 cycles of 15 seconds 95 degC, 30 seconds optimum annealing temperature, 15 seconds 72 degC extension, followed by a dissociation curve with 1 minute 95 degC, 30 seconds at optimum annealing temperature, and dissociation ramp rate at 0.01 degree/second to 95 degC with all points data collection on. qPCR data was analyzed using qBase version 1.3.5 [15]. qPCR product size was assessed with Agilent DNA 1000 Series II (Agilent Technologies) microfluidics chips.

A no reverse transcription control was performed in duplicate using total RNA in the amount to simulate what was used after reverse transcription and amplification from each sample. The TMEM8 3'-most primers were used. All reactions failed. The positive control gave Cts of 22.08 and 22.8.

### Microarray target preparation

For microarray target material used in the smoker vs. nonsmoker portion of the study, 50 ng total RNA was reverse transcribed and amplified per the manufacturer's protocols using the Ovation® RNA Amplification System V2 (Nugen Technologies, Inc.), fragmented and biotin labeled using the FL-Ovation™ cDNA Biotin Module V2, #4200 (Nugen Technologies, Inc.). Gene expression was determined by hybridization of the labelled template to hgU133 Plus 2.0 human microarrays (Affymetrix, Inc., Santa Clara, CA). Hybridization cocktail synthesis and post-hybridization processing was performed according to the "Affymetrix GeneChip Eukaryotic Array Analysis" protocol found in the appendix of the Nugen protocol for the fragmentation kit. Arrays were hybridized for 18 hours and washed using fluidics protocol FS450_0004 on a GeneChip Fluidic Station 450 (Affymetrix, Inc.)

### Microarray pre-processing

Quality assessment of the arrays was performed with the tools available in the Gene Chip Operating Software, version 1.4 (Affymetrix, Inc.) and the Bioconductor packages AffyQCReport [16] and AffyPLM [17], R version 2.8, Bioconductor version 2.3 [18]. The microarray data has been assigned series number GSE16149 in the Gene Expression Omnibus (GEO, http://www.ncbi.nlm.nih.gov/geo/).

### Microarray data analysis

Array data were processed with Robust Multiarray Average (RMA) [19] and quantile normalized using the package available at the Automated Microarray Pipeline (AMP) [20]. Differential expression analysis comparing smokers to nonsmokers was performed with both Significance Analysis of Microarrays (SAM) [9] and Rank Product Analysis (RP) [10], selected for their different statistical approaches. For RP analysis, samples matching the two poor quality arrays were removed as this analysis utilizes the ranked expression values from replicate samples. This left 12 arrays, six in each replicate group, a and b, for this analysis. Unsupervised hierarchical clustering, T-tests with multiple testing correction, SAM and RP were performed using the packages available in the MultiExperiment Viewer, version 4.3.01 (MeV) [21,22] with default settings. Gene Set Enrichment Analysis, GSEA version 2.04 [8,23] was used to test the array data for enrichment of differentially expressed genes. The default settings were used except the minimum size for gene sets was decreased to ten to allow analysis against the RP_downSm list which GSEA reduced from 17. The same microarray differential expression analysis pipeline was used on the data from series GSE8987 from the GEO database [6], which were designated either "mouth", "never smokers" or "current smokers".

The output gene lists of differentially expressed genes from RP and SAM were evaluated for biological significance using Ingenuity Pathway Analysis, IPA (Ingenuity Systems, Inc., Redwood City CA), for a core analysis. Promoter Analysis and Interaction Network, PAINT version 3.6 [12] analysis using the TRANSFAC public database [13] was used with the same gene lists examining both strands to 2000 bases upstream looking for transcription factor binding sites and summing in TREs any potentially co-regulated genes.

## Results

### Quality assessment

**Figure 1:**
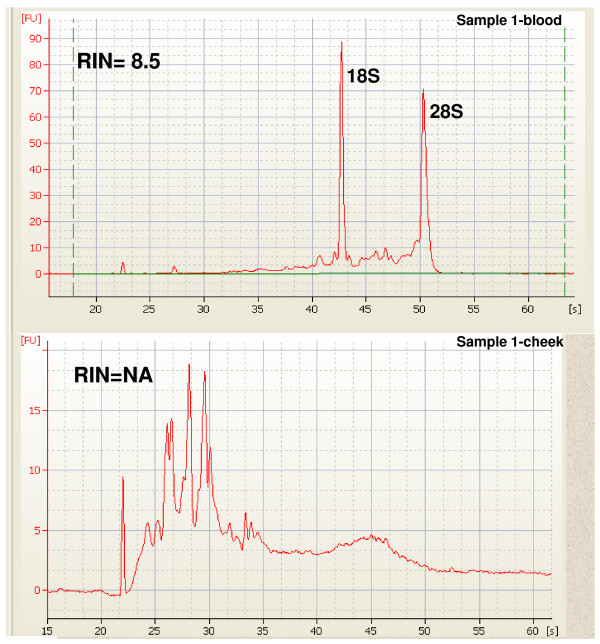
**Representative qPCR matched blood and buccal mucosa samples**. The buccal RNA (cheek) appears to be heavily degraded compared to the blood RNA since there is no evidence of 18 or 28S rRNA peaks and the bulk of material is migrating rapidly indicating small size. RIN, RNA integrity number; NA, no RIN determination possible.

Initially, we determined the quality of RNA purified from buccal mucosa. Matched blood and buccal total RNA from seven subjects was purified (Materials and Methods). RNA quality was assessed on the Agilent Bioanalyzer RNA using Nano 6000 chips (Figure 1). Buccal RNA samples were found to be severely degraded with RNA Integrity Numbers (RINs) routinely less than three. In contrast, RINs from the blood samples were greater than seven in all cases (Additional file 1). These results indicate that buccal RNA was not of high quality.

### Evaluation by qPCR

To determine if RNA from buccal cells could be useful for marker analysis, we chose to perform qPCR on these paired samples. Primers to four genes were used: ITGA5, ANKRD28, TMEM8, and RPS3A. Primers to these genes had been designed previously by our group for another qPCR study and were found to yield detectable signal using total RNA from blood (unpublished results). BioGPS [24] values for these four genes indicated an approximate expected ratio of buccal cells (salivary gland used for estimate) versus blood (Table 2). These four genes appeared to represent a range of mRNA prevalence in salivary gland which we felt was useful in determining our qPCR limitations with the buccal material. Primers were designed to the 3' ends of all four genes. To determine whether RNA degradation was random or specific by gene region, primers to upstream regions of ITGA5, ANKRD28 and TMEM8 were also designed (Additional file 2).

**Table 2:** BioGPS expression values for blood and salivary gland for genes tested via qPCR

	**ANKRD28**	**TMEM8**	**RPS3A**	**ITGA5**
	
Salivary gland	130	2000	60,000	2000
Blood	130	7000	> 100,000	7000

Values are approximate signal strength values from BioGPS using the Human U133A gcRMA dataset [24].

The WT Ovation Pico kit containing random primed hexamers and poly-T primers was used for amplification of all fourteen samples, both blood and buccal, and the subsequent product used for qPCR with the primer pairs detailed above. An average over the seven subjects showed that there was a lower apparent transcript copy-number for each tested gene in buccal mucosa RNA than in blood RNA (Table 3). In some subjects, no Ct was calculated and the differences between apparent transcript levels are greater than the mean value indicates. As seen from the increased standard deviations, RNA from buccal cells had greater variability in Cts, suggesting that buccal RNA quality is more variable than blood RNA.

**Table 3:** qPCR results comparing blood and buccal RNA across four genes with WT amplified template

	**TMEM8**				**ANKRD**				**ITGA5**			**RPS3A**
							
	_2067*	_1372	_584		_4387	_2552	_1345		_3879	_1994		_198
Subject												
Buccal 1	33.76	No Ct	34.93		No Ct	30.33	28.91		31.7	No Ct		31.86
Buccal 2	29.72	29.15	26.83		31.64	29.52	27.47		No Ct	No Ct		28.65
Buccal 3	28.18	30.52	29.27		30.81	35.73	30.13		No Ct	38.64		28.49
Buccal 4	24.96	27.74	28.09		31.8	33.55	31.98		35.71	No Ct		26.82
Buccal 5	25.31	26.56	26.39		31.36	32.8	34.02		No Ct	No Ct		27.38
Buccal 6	35.22	34.85	34.1		No Ct	No Ct	37.42		No Ct	No Ct		ND
Buccal 7	29.05	29.24	35.31		No Ct	No Ct	No Ct		No Ct	No Ct		33.19
**Mean**	**29.46**	**29.68**	**30.7**		**31.4**	**32.39**	**31.66**		**33.71**	**38.64**		**394**
**StDev**	**3.61**	**2.63**	**3.65**		**0.38**	**2.24**	**3.32**		**2.01**	**0**		**2.33**
												
Blood 1	27.03	26.49	25.78		30.58	29.28	30.36		24.86	26.67		26.31
Blood 2	28.5	28.28	26.58		29.04	28.87	28.86		24.41	26.42		26.66
Blood 3	28.08	27.71	26.7		29.03	29.93	28.33		24.28	25.34		26.1
Blood 4	27.64	26.53	26.57		29.43	29.61	29.68		25.14	25.62		27.11
Blood 5	27.3	27.63	25.32		29.82	28.71	28.89		24.72	25.29		26
Blood 6	27.96	27.67	26.02		29.68	29.09	29.45		26.69	28.92		ND
Blood 7	27.8	28.48	27.62		29.49	28.73	28.06		24.34	27.14		26.82
**Mean**	**27.76**	**27.57**	**26.37**		**29.57**	**29.17**	**29.09**		**24.92**	**26.49**		**26.5**
**StDev**	**0.46**	**0.78**	**0.69**		**0.48**	**0.43**	**0.74**		**0.78**	**1.19**		**0.4**
Controls^												
NTC	39.4	No Ct	No Ct		No Ct	No Ct	No Ct		No Ct	No Ct		No Ct
WT amplification	28.4	27.28	26.45		29.47	29.23	29.48		24.63	26.59		2594
3' amplification	26				22.9				24.4			20.5
												
Product (bp)	179	110	118		199	150	143		104	107		276
mRNA (bp)	2529	2529	2529		6339	6339	6339		4248	4248		903

Results are in Ct values from the four genes used in initial expression assessment by qPCR. Results shown are the average value from three replicates for each primer pair as input material from each subject.

* Primers used in qPCR are given by gene heading followed by 5' position of amplification on mRNA. See Additional file 1.

^ NTC, No template control; WT whole transcriptome, and 3' Positive Controls are RNA from pooled blood samples

(ND, not done; bp, basepair)

When specificity of degradation was investigated, no clear pattern was evident. ITGA5 showed a 32-fold difference from 5' to 3' in buccal mucosa compared to an approximately three-fold difference in blood, but most reactions with ITGA5 primers with buccal RNA failed. ANKRD28 and TMEM8 showed no change in 5'/3' ratio in either RNA source. Due to the short transcript length of RPS3A, no 5' primer set was designed. This initial analysis of the quality of buccal RNA shows that, in general, there were lower but detectable levels of target mRNA in buccal mucosa when compared to blood (Table 3). These results do not differentiate between tissue-specific expression differences or degradation; however, when the expression data from BioGPS and the RINs are factored into our analysis, the differences in Cts are greater than expected from expression data and likely due to degradation. The variability of results from buccal cells suggests that the degradation seen in the buccal samples is not occurring in a predictable directional fashion but randomly such that transcript size has no clear effect on the level of degradation seen.

The reduced signal detected in buccal versus blood samples with the WT amplification method led us to hypothesize that a 3'-specific amplification would increase the sensitivity of expression assays by increasing the specificity of the reverse transcription step for mRNAs. With the level of degradation found in buccal samples, rRNA peaks are not detectable in electropherograms and presumably would be reduced in their normal high degree of secondary structure. The degraded rRNA would be accessible to the random primers used in whole transcriptome amplification; however, the poly-T primers used in the 3' amplification approach would not anneal to the fragmented rRNA but be specific for polyA tails of mRNA. This would result in a difference in Ct between the template types, higher when a large percent of the cDNA is ribosomal in origin as opposed to lower Ct values when 3' amplified material is used. To investigate this possibility, the same samples were amplified with the Ovation RNA Amplification System V2, a 3' specific method. Table 4 shows a comparison of the qPCR results using the 3' targeted primers and both buccal mucosa and blood derived RNA template. For all three genes 3' amplification resulted in a Ct decrease, i.e. an apparent increase in copy number, although Cts from buccal mucosa RNA tested with primers to ITGA5 remained greater than 31. Relative Cts from ANKRD28 and TMEM8 between buccal RNA and blood RNA compare favourably with data from BioGPS comparing salivary gland to whole blood. However, ITGA5 values did not correspond particularly well suggesting that ITGA5 was more sensitive to degradation than the other genes tested or than salivary gland data in BioGPS is not predictive for buccal mucosa.

**Table 4:** qPCR results comparing methods of template amplification

Template	Itga5b 3'	Itga5b WT	Tmem8 3'	Tmem8 WT	Ankrd28 3'	Ankrd28 WT
Buccal 4	31.25	39.15	20.38	24.96	20.68	31.8
Buccal 5	35.81	No Ct	20.36	25.31	20.92	31.36
Blood 4	21.43	23.96	21.87	30.68	20.33	29.43
Blood 5	20.56	23.9	21.11	30.23	20.22	29.82
Control	21.18	23.8	21.63	28.4	20.23	29.47

All amplifications were performed using either the Ovation V2 (3') or WT pico Nugen Kits, see Materials and Methods. Control RNA is from a pooled blood sample, see Materials and Methods.

### Microarray study

**Figure 2:**
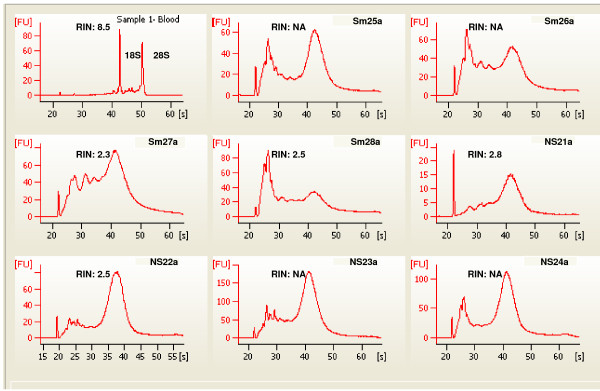
**Buccal mucosa total RNA from smokers and nonsmokers, the group a buccal cell samples**. Note variation between the isolates in peak heights and species. Sm Smokers, NS nonsmoker. Sample 1, whole blood total RNA as seen in Figure 1 for comparison, showing 18S and 28S ribosomal peaks. RIN, RNA integrity number; NA no RIN determination possible.

**Figure 3:**
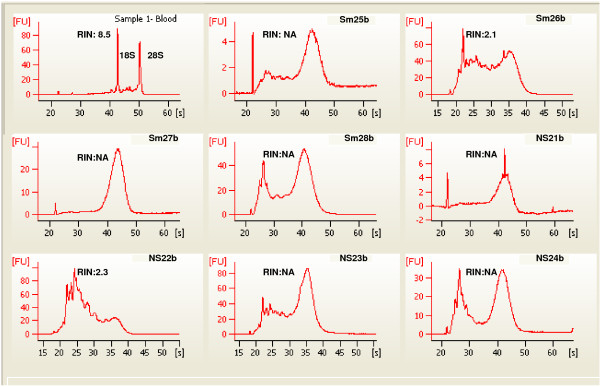
**Buccal mucosa total RNA from smokers and nonsmokers, the group b buccal samples**. Sample 1, total RNA from whole blood is added for comparison. Compare to Figure 2. For example Sm26a and Sm26b are from opposite cheeks of same subject and show some similarity in migration pattern. The same variation in peak heights and species between samples is seen here as in Figure 2. RIN, RNA integrity Number; NA, no RIN determination possible.

Our ability to detect expression of genes by qPCR, most at levels well above background, in 3' amplified samples led us to hypothesize that buccal samples could be used for differential expression testing by microarray analysis. Amplification of buccal RNA samples eliminates the need for repeated sample collection and/or pooling of material from multiple collections. The work of others [5,6] led to the further hypothesis that a comparison of smokers and nonsmokers was a model system likely to allow detection of differentially expressed genes. Affymetrix hgU133 plus 2.0 arrays were used for a global evaluation of gene expression changes between four smokers and four nonsmokers. Only female subjects were used to prevent any gender bias in the data and both cheeks from each subject were sampled. Additionally, an unpaired t-test was performed that showed no statistical significance between the two subject groups based on age (p-value = 0.3737). Total RNA was isolated and evaluated for quality as for the qPCR samples. One cheek sample from each subject was arbitrarily assigned to one of two groups, a or b (Materials and Methods). Figures 2 and 3 show the BioAnalyzer traces from all 16 samples along with a trace representative of the RNA quality usually purified from blood. As seen with the samples used in the qPCR study, the samples show no evidence of rRNA peaks, and a range of degradation product sizes; in only a third of the samples could a RIN be calculated (Additional file 1).

### Quality assessment of the arrays

**Figure 4:**
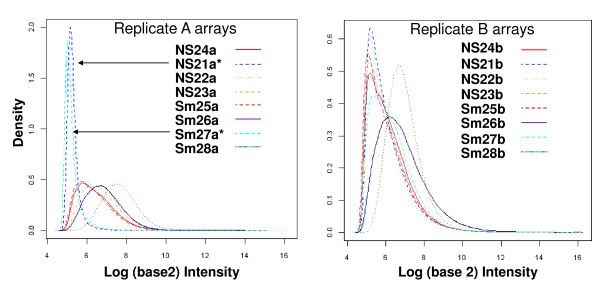
**Replicate Samples a and b Raw Signal Value Histograms**. Two arrays, NS21a and Sm27a have low overall signal strength as shown by the intensity plot. The arrays from the matching b cheek NS21b and Sm27b show acceptable values. Note the difference in y-axis density scale. NS nonsmoker, Sm smoker.

Following hybridization each array was examined for quality. Table 5 lists the percent present (%p) and scaling factor (SF) values determined using the Gene Chip Operating Software (Affymetrix, Inc.; Materials and Methods). Two arrays, NS21a and Sm27a had remarkably low %p and especially high SFs, both indicators of arrays with suspect data quality. Additionally, the same two arrays had much lower signal intensities (Figure 4). The normalized unscaled standard error (NUSE) [25] calculations had high median values and large interquartile range for these two arrays (Table 5). Samples from the same subjects' opposite cheek did not show the same set of quality control issues, further evidence that RNA quality from buccal cells is inconsistent. Neither sample could have been predicted to be of lesser quality from the BioAnalyzer traces (Figures 2 and 3). Due to the poor quality of these two arrays, they were removed from further analysis. Two other arrays, Sm28a and b, had elevated NUSE parameters compared to other subjects but did not have a low %p or high SF, and so were not removed as the observed differences were likely subject dependent and are more likely due to biological diversity between subjects.

**Table 5:** Microarray quality metrics

**Samples**	**Scaling Factor**	**% Present**	**NUSE Median**	**NUSE IQR**
Smokers				
25a	24.6444	35.9	0.989	0.021
25b	4.532	47.8	0.991	0.021

26a	19.022	31.8	0.989	0.02
26b	3.451	30	1.013	0.04

27a	**255.647**	**6**	**1.101**	**0.089**
27b	3.713	49.9	0.985	0.021

28a	12.806	22.5	1.027	0.046
28b	4.674	24	1.061	0.073

Nonsmokers				
21a	**307.934**	**3.5**	**1.12**	**0.097**
21b	6.852	35	1	0.024

22a	22.185	40.7	0.998	0.019
22b	4.808	47.6	0.993	0.022

23a	20.057	33.6	0.987	0.02
23b	4.689	44.5	0.991	0.022

24a	21.886	39.5	0.988	0.02
24b	3.926	50.9	0.988	0.021

In bold, values indicating poor quality array. Scaling Factor and % Present were determined with GCOS. NUSE, [25] was determined by probe level modelling using the Bioconductor AffyPLM package, see Materials and Methods; IQR, interquartile range.

### Microarray data analysis for differential expression

A study using Affymetrix hgU133A arrays to compare gene expression in "current smokers" and "neversmokers" using RNA from buccal mucosa and nasal swabs was published by Sridhar et al. [6]. This group performed an extensive microarray analysis of gene expression in bronchial lavage samples from current smokers, former-smokers and never-smokers and developed a list of 314 genes differentially expressed in smokers in this tissue [7,26]. Using Gene Set Enrichment Analysis (GSEA), Sridhar examined the smoker buccal and nasal microarray data asking whether the genes on the bronchial-314 gene list showed the same direction of change and identified three leading-edge subsets of genes from the bronchial-314 list which were changing expression in the buccal or nasal data in the same direction as in the bronchial data. These were a 74 gene subset up-regulated in buccal mucosa of smokers, a 120 gene subset up-regulated in the nasal mucosa of smokers and a 50 gene subset down-regulated in nasal mucosa of smokers. The buccal microarray cel files were downloaded from GEO and analyzed in parallel with the data from the current study (Materials and Methods). Initially, unsupervised hierarchical clustering was performed with the summarized data from the current study, termed SmvsNS, and BuccalCompare for the Sridhar study. Neither dataset showed any pattern of clustering by replicate sample (a vs. b) in the case of the SmvNS data, nor by smokers and non-smokers in either dataset.

T-tests comparing the a samples to the b samples in the SmvsNS data were done to evaluate the within-subject variability. There were 871 significant probesets out of 53,800 or 1.62%. Comparing smokers to nonsmokers using the same test gave 178 probesets or 0.33%. Applying a t-test to the BuccalCompare data gave 65 differentially expressed probesets comparing never smokers to current smokers and 66 probesets when a random grouping of odd numbered arrays against even was compared. Taken together, these results suggest that there is at least as much or greater variability among subjects than smoking introduces between the two subject types.

SAM [9] and RP [10] were used to develop lists of differentially expressed genes between smokers and nonsmokers. With the SmvNS data, SAM returned 30 significant probesets with a Q value of 0 at a 10% FDR. All 30 probesets were up-regulated in smokers. For the BuccalCompare dataset there were no significant results from the SAM analysis. With RP analysis of the SmvNS data seventeen genes were found to be down-regulated and 118 genes up-regulated in smokers (Additional file 3). RP analysis could not be performed on the BuccalCompare dataset since there were no replicates.

**Figure 5:**
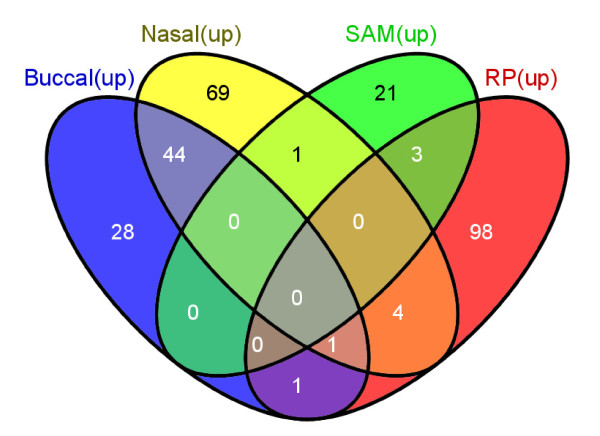
**Venn diagram showing overlap among the four gene lists upregulated in smokers**.

Only a few genes were found to be in common between the up-regulated gene lists (Figure 5) [27]. The RP_downSm gene list had no overlap with the corresponding Sridhar Nasal_downSm leading edge set. Note that the probesets for the genes on the SAM_upSm and the RP_upSm lists have similar fold change ranges and medians, but probesets in the RP_downSm differed in having overall low signal strength (Additional file 4).

Using a similar analysis approach to Sridhar, both the SmvsNS and the BuccalCompare datasets were compared against six gene lists in a GSEA enrichment analysis. The gene lists were the 74 genes in Buccal_upSm, the 120 genes in Nasal_upSm and the 49 genes in Nasal_downSm defined as leading edge subsets by Sridhar [6], the 25 genes in SAM_upSm, the 107 RP_upSm genes, and the 17 genes in RP_downSm the three lists from the current study (Additional file 3).

When GSEA analysis of the SmvsNS dataset was performed against all six gene lists, the four lists up-regulated in smokers showed the same expression patterns in the SmvsNS dataset, and the two down-regulated gene lists likewise were down-regulated in the SmvsNS dataset. The same analysis was performed using the BuccalCompare data against the same six gene lists with the same results. This showed correlation between the SmvsNS and BuccalCompare datasets in terms of the direction of gene expression change for genes in the six sets. In the SmvsNS comparison the SAM_upSm list genes were significantly enriched in the smoker phenotype with an FDR q-value 0.029 and p-value 0.025 but not the RP_upSm genes which showed an FDR q-value 0.3. This was unexpected since the RP_upSm gene list was derived from the SmvsNS dataset. The BuccalCompare data behaved similarly with only the Buccal_upSm gene list statistically significantly enriched. This was expected since it was derived from this dataset.

As a check for reproducibility, two subjects, one smoker and one nonsmoker, both cheeks, were retested several months after the initial sampling was performed. Four arrays were generated (11Sm a, b and 12NS a, b). This small dataset was examined with GSEA against the same six gene sets. The results showed that this repeated subset had significant gene enrichment for smokers with the RP_upSm, Nasal_upSm and Buccal_upSm gene lists with a nominal p-value of 0, an indication of good reproducibility.

### Function analysis

To further evaluate the gene lists derived from the SmvsNS dataset for biological coherence the SAM and RP gene lists were evaluated for over-representation of transcription factor binding sites in the promoters of these genes using the Promoter Analysis and Interactive Tool Set, (PAINT) [12,28], Materials and Methods, and for shared functional interactions using Ingenuity Pathways Analysis, (IPA) (version 7.0, Copyright 2009 Ingenuity Systems, Inc., Redwood City CA). Statistically significant transcriptional regulation elements (TREs) were found with 15 of the SAM_upSm and 42 RP_upSm genes. No TREs were found for genes in the RP_downSm genes.

In IPA, 17 of the 25 genes from SAM_upSm formed two initial networks sharing broad functional categories including tumor morphology, lipid metabolism, carbohydrate metabolism, and small molecule biochemistry that could be merged into a single large network. The RP_downSm genes did not result in any functional networks when examined in IPA. However, 91 of the genes on the RP_upSm list fell into five networks which could be merged into a single large network, indicating shared function. Functional categories for this network included: cell growth, movement, development and death; cell cycle; gene expression, cancer and immunological system development and function.

**Figure 6:**
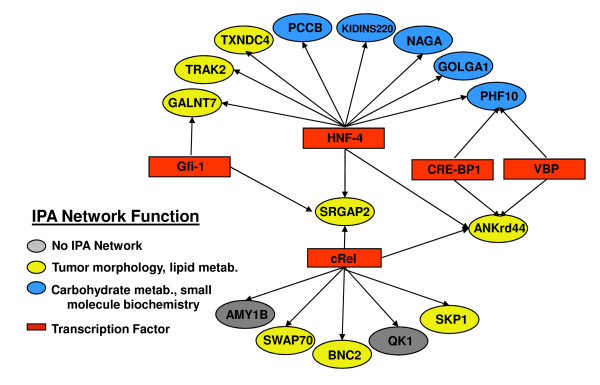
**A graphic showing the PAINT TRE for the SAM_upSm gene list**. Color has been added to indicate membership in a particular IPA functional network. Thirteen of the 25 SAM_upSm target genes are contained in both IPA and PAINT analyses. The ovals represent target genes identified by PAINT as having transcription factor binding sites upstream of the gene. The color of the oval corresponds to the functional networks in which IPA placed the gene. The two gray ovals represent genes not included in the IPA network. The rectangles indicate transcription factors. Arrows connect the transcription factors to genes with corresponding upstream binding sites.

**Figure 7:**
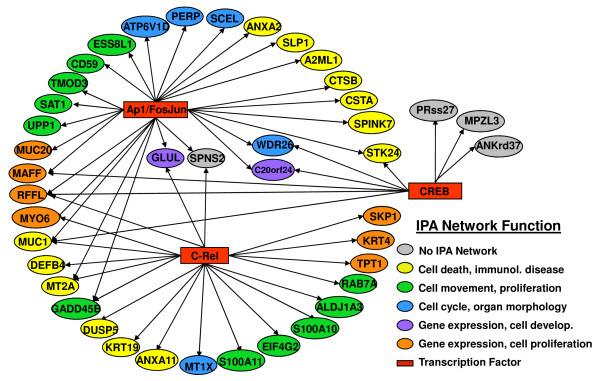
**A graphic showing the PAINT TRE for the RP_upSm gene list**. Thirty-eight of 103 RP_upSm target genes are contained in both IPA and PAINT analyses. The ovals represent target genes identified by PAINT as having transcription factor binding sites upstream of the gene. The color of the oval corresponds to the functional networks in which IPA placed the gene. The four gray ovals represent genes not included in the IPA network. The rectangles indicate transcription factors. Arrows connect the transcription factors to genes with corresponding upstream binding sites. Compare to figure 6.

As a final step in the analysis, genes in TRE networks from PAINT were coded for network function from IPA (Figure 6 and 7). This analysis strongly suggests co-regulation within functional networks and speaks to the transcriptional affects of smoking on buccal cells.

## Discussion

This study was focused on determining whether the buccal mucosa could serve as a tissue source for total RNA to be used in relative gene expression studies and biomarker detection by qPCR and microarray analyses. Two previous studies had suggested that buccal cells had efficacy for measuring responses to tobacco smoke exposure [5,6] and suggested extrapolation of this tissue source to other inhalation or ingestion exposures [5].

Our initial RNA isolations from matched blood and buccal RNA showed a marked difference in the quality of the isolated material between the two sources and showed that there was significant degradation in buccal mucosa RNA. The qPCR results from the matched samples showed an average lower copy number in buccal RNA than blood RNA for all four genes tested and greater variability between subjects (Table 3). The lower copy-number was expected as salivary glands express all four genes at the same or lower level as blood on microarrays (Table 2); however, the increased variability found between buccal samples, including duplicate samples from the same subject over blood is a concern.

The amplification protocols utilized here allow buccal cell samples to be used in repeated measures experiments removing the necessity to sample more than once to obtain sufficient template for a single microarray. The fifty nanograms of RNA we used for amplification can routinely be isolated from a single swab (Additional file 1) and the resulting amplified cDNA is sufficient for an array as well as other procedures such as qPCR. This is in contrast to the multiple sampling and pooling from the same individual required by Sridhar et al. [6] where amplification was not used. Additionally, there was an advantage to using 3'-amplification over a whole transcriptome approach with the degraded buccal RNA possibly due to a reduction in the rRNA contribution to the amplified product.

In most cases, the array quality was acceptable but with buccal RNA, arrays did have a higher failure rate than is typical for arrays hybridized with target material from blood RNA. Two of 16 samples failed where matching samples from the other cheek passed. This opens the possibility that samples from both cheeks would be required to insure that every sample was collected in a study. However, we found the intra-subject variability to be high as well. The availability of the Sridhar buccal dataset provided comparison data and along with the previous work from this group [7], also provided published lists of genes from buccal and nasal cells which change expression levels due to smoking. Gene lists developed from the current study did not overlap extensively with each other or with the Sridhar lists. However, using the independent analysis tools PAINT and IPA a cohesive function/cotranscription network was generated suggesting two non-random sets of genes upregulated in smokers. Transcription factor binding site analysis is a good complement to a functional analysis such as IPA because it has no *a priori *assumptions about gene function relying instead on promoter sequence alone. The analysis results suggested that using an approach which included these two complementary methods is useful for evaluating candidate genes.

The analysis conducted with GSEA was significant because there was perfect concordance between gene lists derived from each of the two datasets for the direction of change in expression between smokers and non-smokers. The results from the small repeated dataset were an indication of reproducibility with this system. This validated the methods used in the current study to discover differentially expressed genes. However, the lack of consistent statistically significant enrichment for the smoker phenotype with GSEA analysis taken with the degradation in RNA derived from buccal cells highlight the difficulties to be expected when using buccal-cell RNA for differential expression testing.

## Conclusions

The work presented here was a straightforward evaluation of buccal mucosa as a tissue useful for evaluating relative gene expression changes using an analysis scheme containing well validated and commonly used analysis tools. Isolation and amplification techniques were successfully modified from those used with whole blood. The level of degradation found was not unexpected; nevertheless, we were able to successfully perform qPCR with the buccal RNA. Somewhat surprising was that, given the poor quality of the RNA, the quality of the majority of the microarrays was acceptable and that several lists of genes showing change in expression in smokers compared to nonsmokers resulted from statistical analysis of the arrays. There was evidence of reproducibility in expression change but the borderline statistical significance level of the lists clouds the validity of the findings. Our findings suggest that this may be a difficult tissue to use, requiring replicate sampling and arrays, which may perform better using a different technology such as an array format or amplification method designed for heavily degraded template material. However, using buccal tissue RNA with 3' amplification may be a suitable tissue choice and preparation approach when assaying specific highly differentially expressed gene targets which could overcome the limitations of subject variability and sample degradation.

## Competing interests

The authors declare that they have no competing interests.

## Authors' contributions

DMK participated in design of study, performed data analysis, and drafted the manuscript, VLW isolated RNA and performed amplifications, MCJ performed qPCR and microarray hybridizations, DB conceived the study and reviewed the manuscript. All authors have read and approved the final manuscript.

## Pre-publication history

The pre-publication history for this paper can be accessed here:

http://www.biomedcentral.com/1755-8794/3/24/prepub

## Supplementary Material

Additional file 1 **Total RNA yields and quality from blood and buccal samples used in study**. Click here for file

Additional file 2 **Primers used for qPCR portion of study**. Click here for file

Additional file 3 **Gene lists used for the GSEA analysis**. Click here for file

Additional file 4 **Fold change of probesets included in the RP and SAM gene lists**. Click here for file
